# *Helicobacter pylori* Infection in Endoscopic Biopsy Specimens of Gastric Antrum: Laboratory Diagnosis and Comparative Efficacy of Three Diagnostic Tests

**DOI:** 10.1155/DTE.6.25

**Published:** 1999

**Authors:** G. M. Malik, M. Mubarik, S. A. Kadla

**Affiliations:** 1 Department of Medicine SMHS Hospital Government Medical College Srinagar – 190010 Kashmir India; 2 Nalbandpora Safakadal Srinagar – 190002 Kashmir India

## Abstract

*Aims and objectives* The present study was undertaken to compare the diagnostic yield of three available test procedures for detecting *Helicobacter pylori* (*H. pylori*) infection in endoscopic biopsies.

*Methods H. pylori* infection was sought in 150 patients referred for upper gastrointestinal (GI) endoscopy. Multiple (about six) biopsy specimens were taken from pyloric antrum in each patient. Two biopsy specimens were subjected to one minute endoscopy room test – OMERT (a modified form of urease test), two were sent for histopathological analysis, where multiple sections were subjected to Giemsa staining and two were sent for microbiological evaluation after Gram's staining of heat fixed biopsy material.

*Results H. pylori* positivity using histology, microbiology and OMERT was observed to be 33%, 30% and 27% respectively. However, overall 40% patients were infected when the results from three test procedures were combined, as *H. pylori* positivity was repeated more than once by these procedures separately. Histology was found to be superior to other two tests in our study, especially when multiple sections were examined, for the distribution of the organism was patchy. Amongst the infected, *H. pylori* was seen in only 30% of all 3–8 sections cut from a biopsy, whereas in 70% it was noted in a single section only.

*Conclusion* The study revealed that histology has the highest detection rate and can be chosen as the “gold standard” amongst the three low cost test procedures available at present in our setup.


*Diagnostic and Therapeutic Endoscopy*, Vol. 6, pp. 25–29
Reprints available directly from the publisher
Photocopying permitted by license only

© 1999 OPA (Overseas Publishers Association) N.V.
Published by license under
the Harwood Academic Publishers imprint,
part of The Gordon and Breach Publishing Group.
Printed in Malaysia.

### *Helicobacter pylori* Infection in Endoscopic Biopsy Specimens of Gastric Antrum: Laboratory Diagnosis and Comparative Efficacy of Three Diagnostic Tests

G.M. MALIK, M. MUBARIK* and S.A. KADLA

*Department of Medicine, SMHS Hospital, Government Medical College, Srinagar – 190 010, Kashmir, India*

*(Received 25 February 1999; Revised 8 June 1999; In final form 17 June 1999)*

***Aims and objectives*** **The present study was undertaken to compare the diagnostic yield of three available test procedures for detecting *Helicobacter pylori* (*H. pylori*) infection in endoscopic biopsies.**

***Methods*** ***H. pylori*** **infection was sought in 150 patients referred for upper gastro-intestinal (GI) endoscopy. Multiple (about six) biopsy specimens were taken from pyloric antrum in each patient. Two biopsy specimens were subjected to one minute endoscopy room test – OMERT (a modified form of urease test), two were sent for histopathological analysis, where multiple sections were subjected to Giemsa staining and two were sent for microbiological evaluation after Gram's staining of heat fixed biopsy material.**

***Results*** ***H. pylori*** **positivity using histology, microbiology and OMERT was observed to be 33%, 30% and 27% respectively. However, overall 40% patients were infected when the results from three test procedures were combined, as *H. pylori* positivity was repeated more than once by these procedures separately. Histology was found to be superior to other two tests in our study, especially when multiple sections were examined, for the distribution of the organism was patchy. Amongst the infected, *H. pylori* was seen in only 30% of all 3–8 sections cut from a biopsy, whereas in 70% it was noted in a single section only.**

***Conclusion*** **The study revealed that histology has the highest detection rate and can be chosen as the "gold standard" amongst the three low cost test procedures available at present in our setup.**

*Keywords:* Biopsy, Endoscopy, *H. pylori*, Histology, Microbiological, Urease

### INTRODUCTION

In 1983, Warren and Marshall reported unidentified curved bacilli in the gastric antral biopsies from patients with active gastritis and peptic ulcer disease [1]. This bacteria was *Campylobacter pylori.* Subsequently its name was changed to *Helicobacter pylori* [2,3]. Currently there are many diagnostic tests for detection of infection with this organism, but there is no commonly acknowledged "gold

* Corresponding author. Nalbandpora, Safakadal, Srinagar – 190 002, Kashmir, India.

standard" method for diagnosing *H. pylori* infection. The best way to detect *H. pylori* in histological sections would be to examine suitably stained sections of a biopsy specimen under high power of microscope. In addition to this, the other methods like urease test and microbiological evaluation also establish the diagnosis of *H. pylori* infection [4]. Not much work has been carried out on the comparison of these three available test procedures from the Indian subcontinent and this is the first study of its kind reported from the Kashmir valley.

The aims of our study were: (1) to compare the diagnostic yield of above three low cost and available test procedures in detecting *H. pylori* infection in endoscopic biopsy specimens of gastric antrum; (2) to determine which test amongst the three is the most specific and sensitive and can be taken as the "gold standard"; and (3) to determine *H. pylori* status in relation to histological findings by the above tests.

### MATERIALS AND METHODS

The study comprised 150 patients referred for routine upper gastrointestinal (GI) endoscopy. Their mean age was $34.80 \pm 10.19$ years with 125 males and 25 females. The main indication for biopsy was dyspepsia. Patients who had taken antibiotics, $H_2$ blockers, colloidal bismuth or omperazole one to two months prior to endoscopy were not included in the study [5,6]. From each patient six biopsies were taken from the gastric antrum within 5 cm of pylorus, as antrum appears to be more uniformly involved in *H. pylori* infection [7,8]. These biopsy specimens (two chips each) were subjected to following test procedures.

### One Minute Endoscopy Room Test (OMERT)

In this test, two biopsy specimens were put in 1 ml of 10% w/v freshly prepared urea solution in deionized water (pH 6.8) at room temperature. Two drops of 1% phenol red were added to above solution as an indicator. A change in colour from yellow to pink observed 1–5 min after addition of indicator was taken a positive test (i.e. *H. pylori* present), whereas absence of such a colour change or change of colour after 5 min was taken as negative test [9].

### Histological Analysis

Two antral biopsy specimens from each patient were fixed in 10% buffered formalin. Paraffin wax sections were cut after routine processing [4,10,11]. These were stained with May–Grunwald–Giemsa stain [12]. From each biopsy specimen 3–8 Giemsa stained sections were prepared. The entire epithelial surface of all stained sections was examined under oil immersion by one observer who did not know the result of other test procedures. The presence of curved bacilli in the vicinity of gastric epithelium was taken to be indicator of *H. pylori* infection. All the sections of paired biopsy specimens were studied for presence or absence of the organism. Further, the histology of gastric mucosa was also studied in relation to *H. pylori* [4,7,8].

### Microbiological Analysis

Two biopsy specimens were rubbed on a dry glass slide, heat fixed and then stained with Gram's stain, and finally studied for the presence of *H. pylori* under light microscopy [2].

### Ethics

All subjects gave informed consent for the collection of biopsy tissue. Human experimentation guidelines of the "Decleration of Helsinki" were followed.

### RESULTS

Of 150 patients biopsied, overall 60 (40%) were infected when the results of the three test procedures were added. The results were added because *H. pylori* positivity was represented more than once by three test procedures separately. *H. pylori*

postivity was observed as 50 (33%), 45 (30%) and 40 (27%) using histology, microbiology and OMERT respectively. Twenty nine (19%) patients were detected positive by both histology and OMERT, 34 (23%) by both histology and microbiology, 32 (21%) by both OMERT and microbiology and 22 (15%) by all the three test procedures separately (Table I).

TABLE I Diagnostic test results in 150 patients biopsied for *H. pylori* infection

| Test procedure | *H. pylori* positive patients | % |
|---|---|---|
| 1. Test procedures in combination* | 60 | 40 |
| 2. OMERT** | 40 | 27 |
| 3. Microbiology (Gram's staining) | 45 | 30 |
| 4. Histology | 50 | 33 |
| 5. Two test procedures positive | | |
| (a) Histology + OMERT | 29 | 19 |
| (b) Histology + Gram's staining | 34 | 23 |
| (c) OMERT + Gram's staining | 32 | 21 |
| 6. All three test procedures positive | 22 | 15 |

*At least one test procedure was positive.
**OMERT (one minute endoscopy room test).

TABLE II Comparative efficacy of diagnostic tests for *H. pylori*

| Variable | Test procedures | | |
|---|---|---|---|
| | Histology | OMERT | Gram's staining |
| Positive | 50 | 40 | 45 |
| Negative | 100 | 110 | 105 |
| False positive | — | 10 | 10 |
| False negative | — | 10 | 05 |
| Sensitivity | — | 75% | 78% |
| Specificity | — | 89% | 86% |

*Note:* The absence of false positives and false negatives in case of histology infer that histology proved to be "gold standard" amongst the three available test procedures (considering its highest detection rate for *H. pylori*) in our study. Further, taking sensitivity and specificity by histology as 100% (standard), the same was observed to be much less in case of OMERT and Gram's staining.

Ten patients detected positive by microbiology and OMERT were negative by histology. On the other hand, 10 patients detected negative by OMERT and 5 by microbiology were positive by histology (Table II). The comparative sensitivity and specificity of histology OMERT and microbiology (Gram's staining) are also represented in this table.

The histopathological examination of the biopsy smears revealed changes of chronic superficial gastritis, chronic active gastritis in some patients, whereas majority of patients had normal gastric mucosa (Table III). The *H. pylori* status in relation to histopathologic changes of gastric mucosa is also revealed in this table.

Thirty percent of the infected patients had *H. pylori* present in all the section of biopsy specimens and the remaining 70% had the organism present in one biopsy section, other sections being negative.

### DISCUSSION

Currently many different diagnostic tests exist for detecting *H. pylori* infection. Each test has its own merits and demerits in terms of indication, sensitivity, specificity, cost and time [5,13]. Many different protocols have been used to detect *H. pylori* in biopsy specimens. The protocols include urease test, histology, microbiology, culture and polymerase chain reaction. Currently polymerase chain

TABLE III Histological findings and *H. pylori* status of biopsy specimens ($n = 150$)

| Histological finding | No. of cases (%) | *H. pylori* positivity (No. (%)) by | | |
|---|---|---|---|---|
| | | Histology | OMERT | Gram's staining |
| Normal gastric mucosa | 100 (67) | 25 (25) | 15 (15) | 15 (15) |
| Chronic active gastritis | 30 (20) | 20 (67) | 25 (83) | 20 (67) |
| Chronic superficial gastritis | 20 (13) | 05 (25) | 05 (25) | 05 (25) |
| Total | 150 | 50 (33) | 45 (30) | 40 (27) |

reaction is experimental and gives lot of false positive results which therefore, limits its use as a gold standard. Culture of the biopsy specimens cannot be used routinely as it is time consuming and is very difficult to maintain the strict anaerobic measures. However, the bacterial culture surely yields high results and provides information about the specific antibiotics to be used for eradicating the bacteria in different patients, keeping in view the development of resistance [3,5,6]. ELISA serology for the diagnosis of *H. pylori* is done by assessing the IgG antibodies and has sensitivity and specificity of 95%. But, the test is costly and has a false positive result of 10% [12,13]. C13 urea and C14 urea breath tests are highly specific (98%) and very sensitive (95%). But these tests are costly and therefore cannot be advocated for routine use. Thus, the choice lies in urease test, histology and microbiology [4,5]. Our study was also aimed at these three low cost test procedures, available in our setup at present.

Urease test detects the urease activity of the organism. Conventional urease test has sensitivity and specificity of 84% and 86% respectively. However, it has been claimed that 5–10% patients have low number of *H. pylori*, which cannot be detected by urease test. Various modifications of the conventional urease test have taken place from time to time simply to increase the sensitivity and specificity. The one such modification is OMERT which have sensitivity of 91% and specificity of 100%. Further, OMERT is cheap compared with the CLO rapid urease test widely mentioned in the literature [3,4,9]. Variations do exist in the methods used to detect *H. pylori* by microbiology. The results are excellent by rubbing the biopsy specimen over a dry glass slide rather than cutting or grinding the biopsy specimen [4].

In our study, however, we found that OMERT and the microbiological analysis of the biopsy specimens were almost equally sensitive and specific but having lower yield of the bacterial detection as compared to histology. Further, both of these procedures do not provide the information about the presence of associated gastritis, which is however, true with histology.

Histology has been used as a diagnostic tool for *H. pylori*. Various stains like haematoxylin and eosin, Giemsa, Gram's, 1% methylene blue, Warthin Starry silver method and fluorescent stains [3]. As per current recommendations, Giemsa staining is the best stain, as it is cheap, less time consuming and diagnostic yield is increased as compared to other staining procedures [13]. Barry J. Marshall advocates "If the *H. pylori* diagnosis is going to make an important contribution to management, then the most sensitive test should be used. Currently this is histology" [13]. This is in confirmity with our observation that histology (Giemsa staining) detected most of the infected patients who underwent gastroscopy in our study. Histology, in addition to detection of the organisms, also helps in establishing the diagnosis of associated gastritis (if present) in the biopsy specimen. We observed the increased association of *H. pylori* with chronic active gastritis (Table III), in confirmity with earlier reports [1,2].

The distribution of *H. pylori* in gastric mucosa is patchy. Therefore, the antral biopsy specimens may not be containing the organisms at all and hence *H. pylori* cannot be diagnosed in such a condition. In accordance with this study we, like others [4,6] also believe that multiple biopsies (at least two) from multiple sites of the stomach, should be taken in order to increase the diagnostic yield. Further, we observed that the chance of detecting *H. pylori* increased when maximum sections of the biopsy specimens were studied, that can again be explained on the basis of patchy distribution of *H. pylori*.

In this study we observed that histology had the highest detection rate, compared to other two tests. Further, histology, unlike the other two tests detected associated gastritis as well. Thus, histology is superior to other test procedures and we also consider it to be "the gold standard" for diagnosis of *H. pylori* infection in confirmity with some earlier reports [4,5,13]. Thus (taking histology as "gold standard") some of the positive and negative observations of the other two tests, not coinciding with histology were considered as false. Why such false observations with these two tests were noticed is not known. Further, taking sensitivity and specificity of histology (being "gold standard") as 100%, the same was much less in case of the other tests.

In conclusion, it was observed that histology was superior to other two test procedures in detecting *H. pylori* infection in antral biopsy specimens and can be taken as "gold standard". However, multiple biopsy specimens with as many sections should be studied to ensure optimal detection.

### *Acknowledgement*

We are indebted to Mr. Emm Afsurdah for computer execution of this manuscript.

### *References*

[1] Warren, J.R. and Marshall, B.L. Unidentified curved bacilli on gastric epithelium in active chronic gastritis. *Lancet* 1983; **1**: 1273–1275.

[2] Jiang, S.J., Liu, W.Z., Zhang, D.Z. *et al. Campylobacter* like organisms in chronic gastritis, peptic ulcer, and gastric carcinoma. *Scand. J. Gasteroenterol.* 1987; **22**: 553–558.

[3] Kyle, E.B. and Dravid, A.P. Diagnosis of *H. pylori* infection. *Gastroenterology Clinics of North America* 1993; **22**(1): 105–115.

[4] Mohammad, A.E., Al-Karawi, M.A., Ahmad, A.M.M. *et al.* Helicobacter pylori: incidence and comparison of three diagnostic methods in 196 Saudi patients with dyspepsia. *Hepato-Gastroenterol.* 1994; **41**: 48–50.

[5] Logan, R.P.H., Polson, R.J., Misiewicz, J.J. *et al.* Simplified single sample 13Carbon urea breath test for *Helicobacter pylori.* Comparison with histology, culture, and ELISA Serology. *Gut* 1991; **32**: 1461–1466.

[6] Dickey, W., Collins, J.S.A., Watson, R.G.P. *et al.* Secretor status and *Helicobacter pylori* infection are independent risk factors for gastroduodenal disease. *Gut* 1993; **34**: 351–353.

[7] Wyatt, J.I., Prinrose, J. and Dixon, M.F. Distribution of *Campylobacter pylori* in gastric biopsies, *J. Pathol.* 1988; **155**: 300A.

[8] Morris, A., Ali, M.K., Brown, P. *et al. Campylobacter pylori* infection in biopsy specimens of gastric antrum: laboratory diagnosis and estimations of sampling error. *J. Clin. Pathol.* 1989; **42**: 727–732.

[9] Arvind, A.S., Cook, R.S., Tabaqchali, S. *et al.* One minute endoscopy room test for *Campylobacter pylori. Lancet* 1988; **26**: 704.

[10] Loffeld, R.J.L.F., Willems, I., Flendrig, J.A. *et al. Helicobacter pylori* and gastric carcinoma. *Histopathology* 1990; **17**: 537.

[11] Mohammad, A.E., Al Karawi, M.A., Al Jumah, A.A. *et al. Helicobacter pylori.* Prevalence in 352 consecutive patients with dyspepsia. *Annals of Saudi Medicine* 1994; **14**(2): 134–135.

[12] Hu, P.J., Mitchell, J.M., Li, Y.Y. *et al.* Association of *H. pylori* with gastric cancer and observations on the detection of this bacterium in gastric cancer cases. *Am. J. Gastroenterol.* 1994; **89**(10): 1806–1810.

[13] Barry, J. and Marshell, M.D. *Helicobacter pylori. Am. J. Gastroenterol.* (Suppl) 1994; **89**(8): S116–S128.

